# Pulmonary artery stiffness assessed by velocity-encoding MRI: comparison of techniques

**DOI:** 10.1186/1532-429X-13-S1-P362

**Published:** 2011-02-02

**Authors:** El-Sayed H Ibrahim, Jean M Shaffer, Richard D White

**Affiliations:** 1 Department of Radiology, University of Florida, Jacksonville, FL, USA

## Introduction

The pulmonary artery (PA) plays an essential role in smoothing the transition from right-ventricular pulsatile flow to the nearly steady-flow at capillary level. The loss of PA compliance has considerable influence on elevated right-ventricle workload. MRI velocity-encoding is an effective technique for assessing pulse-wave-velocity (PWV) by measuring the disturbances in flow or vessel diameter the pressure wave causes [1]. Two methods have been proposed for measuring PWV: transit-time (TT) and flow-area (QA)[2,3]. Nevertheless, no data is available that compares the two methods, especially over wide range of PWV values, or at 3.0-Tesla, which is the purpose of this study.

## Methods

**Figure 1:**
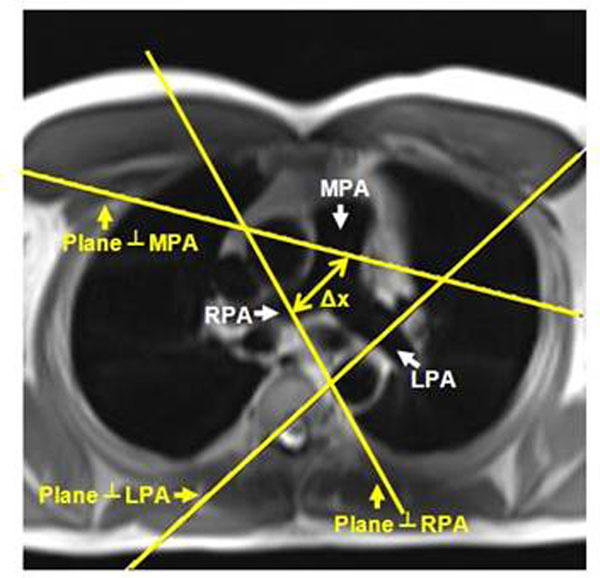
Planning of the pulmonary flow imaging planes. An axial slice showing the main, right, and left pulmonary arteries (MPA, RPA and LPA, respectively). Two planes are prescribed perpendicular to the flow direction in the pulmonary artery. Plane 1 is perpendicular to MPA, while plane 2 is perpendicular to LPA or RPA. The distance along the pulmonary artery between the two measuring sites (Δx) is used in calculating PWV.

**Figure 2:**
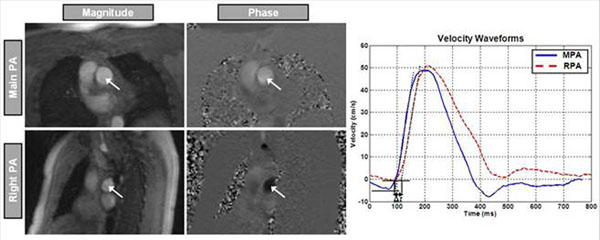
The Transit-Time method for measuring PWV. Anatomical (left) and velocity-encoding (right) images showing cross sections of the main pulmonary artery (up) and right pulmonary artery (down). The curves (right) show the velocity waves at MPA and RPA. The transit time (Δt) is measured between the curves feet. The curve foot is determined as the intersection between the baseline velocity (solid horizontal line) and slope of the up-steeping edge (dotted line). PWV is determined as the ratio of the distance between the two measurement sites (Δx) and the transit time (Δt).

**Figure 3:**
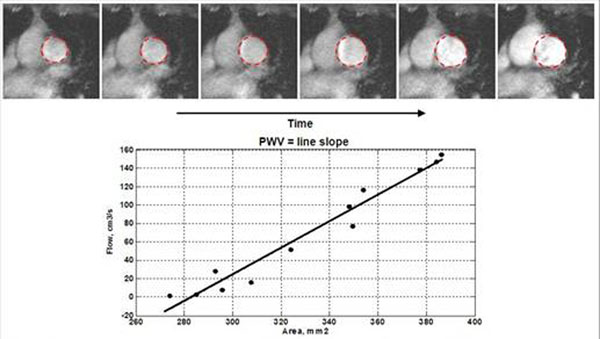
The Flow-Area method for measuring PWV. A succession of cross-sectional images showing the main pulmonary artery distending during early systole. The vessel cross-sectional area and flow are semi-automatically measured for each frame during early systole. The plotting shows the measured areas versus flow. A line is fitted to the data, where PWV is determined as the line slope (flow change over area change).

Twenty-five volunteers(Table 1) were scanned on 3.0-Tesla Siemens scanner. Two velocity-encoding sequences were applied to each subject. The first sequence was optimized for high temporal-resolution (heart-phases=128, pixel-size =1.25mm, venc=150cm/s), and implemented twice: at main PA and either right or left PA locations (Figure 1). The second sequence was optimized for high spatial-resolution (heart-phases=80, pixel-size=0.6mm, venc=150cm/s), and implemented once at the main PA location (Figure 1). The images were analyzed with MATLAB, as previously described for TT and QA methods( Figures 2 and 3) [2,3]. Inter-method, inter-observer and intra-observer variabilities were calculated using Bland-Altman analysis.

**Table 1:** Diversity in the study group (mean ± SD)

Parameter	Mean ± SD
15 males & 10 females	
Age (years old)	52 ± 16
Height (cm)	167 ± 9
Weight (kgm)	82 ± 16
Heart rate (beats per minute)	69 ± 11
Systolic/diastolic blood pressure (mm Hg)	139±14 / 83±10
LV ejection fraction (% )	57 ± 12

## Results

**Figure 4:**
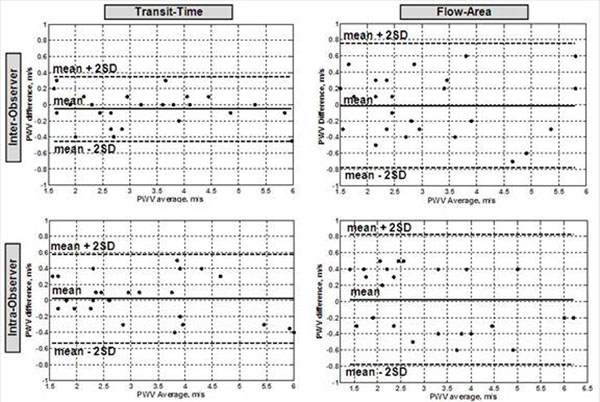
Bland-Altman plots of inter-observer (up) and intra-observer (down) variabilities for the transit-time (left) and flow-area (right) methods for estimating PWV. The data shows low variabilities as all measurement differences lie with the +/- 2SD limit. The flow-area method shows larger variabilities (larger difference standard deviations) than the transit-time method.

The MRI exam lasted for 15-20 minutes. Image analysis lasted for 1 minute and 4 minutes for the TT and QA methods, respectively. The TT and QA methods showed good agreement (P>0.1). The Bland-Altman analysis resulted in mean±SD of 0.13±0.35m/s for the measurement differences. All the differences lied within the ±2SD limit. The correlation coefficient between the two methods was r=0.93. The repeated measurements showed low inter-observer and intra-observer variabilities (Figure 4). The mean±SD of the TT/QA measurement were -0.05±0.2/-0.01±0.38 m/s and 0.02±0.27/0.02±0.4 m/s for inter-observer and intra-observer, respectively. The corresponding correlation coefficients were r=0.96/0.92 and r=0.94/0.90.

## Discussion and conclusions

The TT and QA techniques showed good agreement in estimating PWV, although the QA method resulted in larger variabilities than in TT. Long processing time was required in the QA method, mostly for identifying vessel cross-sections. However, the TT method required double the imaging time as in QA due to the acquisition of two slices. The use of 3.0-Tesla allowed for improving the temporal and spatial resolutions in the TT and QA sequences, respectively. In conclusion, each technique has its own advantages and disadvantages. The choice depends on patient condition, heart rate, and required image quality.
